# Accelerated PARAFAC-Based Channel Estimation for Reconfigurable Intelligent Surface-Assisted MISO Systems

**DOI:** 10.3390/s22197463

**Published:** 2022-10-01

**Authors:** Haoqi Xiao, Honggui Deng, Aimin Guo, Yuyan Qian, Chengzuo Peng, Yinhao Zhang

**Affiliations:** School of Physics and Electronics, Central South University, Lushan South Road, Changsha 410083, China

**Keywords:** channel estimation, reconfigurable intelligent surface, PARAFAC decomposition

## Abstract

To achieve fast and accurate channel estimation of reconfigurable intelligent surface (RIS)-assisted multiple-input single-output (MISO) systems, we propose an accelerated bilinear alternating least squares algorithm (ABALS) based on parallel factor decomposition. Firstly, we build a tensor model of the received signal, and expand it to obtain the unfolded forms of the model. Secondly, we derive the expression of the estimation problem of two channels based on the unfolded forms to transform the problem into a cost function problem. Furthermore, we solve the cost function problem by introducing a simpler iterative optimization constraint and linear interpolation. Finally, we provide a strategy on the receiver design based on the feasibility conditions discussed in this paper, which can guarantee the uniqueness of the channel estimation problem. Simulation results show that the proposed algorithm can obtain a faster estimation speed and less iteration steps than the alternating least squares (ALS) algorithm, and the accuracy of the two algorithms is very close.

## 1. Introduction

Recently, reconfigurable intelligent surface (RIS) is envisioned as a critical technology of 6G wireless communications [1,2,3]. Specially, RIS consists of a large number of reflecting elements, and the wireless environment can be manipulated by adjusting the phase shift and amplitude of the reflective elements [4,5]. Thus, subtly adjusting the reflection coefficient using the RIS can adjust the attenuation and scattering of the incident electromagnetic wave, so that it can transmit in the desired way, which is referred to as a programmable and controllable wireless environment. Since the elements consume little energy, RIS can reduce communication costs without special power amplifiers [6,7]. Therefore, the RIS possesses great potential in enhancing future communication. Accurate and fast channel estimation is necessary for reaching the full potential of RIS. The joint active beamforming at the transceiver and passive beamforming at the RIS needs channel state information (CSI) obtained from channel estimation, and thus channel estimation is a fundamental problem that needs to be solved [8].

Compared with the channel estimation of the multiple-input multiple-output (MIMO) system, the channel estimation of the RIS-assisted MIMO system involves the estimation of multiple channels: the channel between the base station (BS) and the user equipment (UE) and the indirect channel generated by the RIS. In practice, RIS cannot send training sequences independently and the passive elements of the RIS cannot perform active signal processing. Therefore, CSI needs to be acquired at the BS or UE. Furthermore, the introduction of a large number of passive elements in the RIS will cause plenty of unknown channel parameters and require a high estimation overhead. Besides, owing to the nonlinear hardware characteristics of RIS elements, our estimation problem will be more complicated. Hence, channel estimation is a challenging task in RIS-assisted multi-user communication systems. Plenty of work is focused on solving this problem. The authors of [9] proposed that the cascaded channels of the remaining users can be recovered by estimating the cascaded channels of a typical user. In [10], the joint multi-user cascaded channel estimation problem was formulated as a sparse matrix recovery problem, which was solved by subspace projection and iterative reweighting optimization. These algorithms fully consider the channel correlation to estimate the cascaded channels for all users. However, these papers only estimate the cascaded channels. The estimation problem of separate channels still needs to be solved.

Some efforts have been made to solve the estimation tasks for separate channels. In [11], the proposed RIS reflection model allows switching the reflection elements on and off, which can achieve separate channel estimation. The authors of [12] proposed an iterative algorithm to estimate the channel between BS and RIS in the first stage, which required several changes to the RIS phase matrix; in the second stage, the RIS-UE CSI was obtained by performing the least squares (LS) algorithm. However, the application of this method is complicated. These papers have studied the separate channel estimation and proposed some schemes with high estimation accuracy. Yet, these methods will cause higher pilot overhead, since they require changing the reflection elements during the estimation procedure.

Due to the high pilot overhead, many researchers have proposed methods with a lower overhead. The authors of [13] presented a two-timescale channel estimation framework and designed a coordinate descent-based algorithm to estimate the quasi-static BS-RIS channel, then applied the LS-based algorithm to estimate the RIS-UE channel. The estimation scheme requires a low training overhead, but it is not applicable to complex cases. The authors of [14] proposed a two-stage algorithm that includes sparse matrix factorization and complementation. The scheme can achieve accurate channel estimation, yet the performance of the algorithm deteriorates when the channel matrix is at a high sparsity level. In [15], the channel estimation problem was divided into two sub-problems of compressed sensing, which can be solved by applying atomic parametric minimization to find the estimation of the channel parameters sequentially. The sparsity of the channel is also fully considered to reduce estimation overhead, but the selection of the regularization parameters in the algorithm still needs to be optimized.

In addition to passive RIS-assisted MIMO systems, many works have investigated the channel estimation problem in other communication systems. The authors of [16] focused on the RIS-assisted multiple-input single-output (MISO) system and proposed an active sensors-aided channel estimation algorithm to estimate the BS-RIS-UE terahertz channel. The algorithm can accurately estimate the channels with low pilots. In [17], the authors investigated RIS-assisted MISO systems and proposed a linear minimum mean square error (LMMSE) estimator to obtain the CSI for overall channels, and their method can achieve channel estimation with low complexity. The authors of [18] investigated the semi-passive RIS-assisted communication system and proposed a two-stage channel estimation method. In [19], a channel estimation method was proposed utilizing the graph attention network to solve the channel estimation of the RIS-integrated high-altitude platform station. The authors of [20] proposed data-driven non-linear solutions based on deep learning to better approximate the globally optimal minimum mean square error (MMSE) channel estimator, which can achieve superior performance compared with linear estimation approaches.

Although the above methods achieved high channel estimation performance, the computational complexity still needs to be optimized. In recent years, the parallel factor (PARAFAC) decomposition [21,22] has been successfully applied in estimating multiple large channel matrices in MIMO communication systems. It enables low-complexity estimation by decomposing the high-dimensional tensor model of the signal into a linear combination of many rank-one matrices. The authors of [23] proposed a channel estimation method for dual-hop MIMO relay systems, which can effectively reduce the training overhead. To address the current problems of high overhead and complexity of the algorithm in RIS channel estimation, the authors of [24] proposed a simple and effective channel estimation algorithm based on the tensor model of the channel and its algebraic structure. In [25], a vector-based approximate message-passing algorithm was proposed for channel estimation, which is simpler compared to the alternating least square (ALS) algorithm [26]. We summarize the main previous works in Table 1.

Even if the estimation method based on the PARAFAC decomposition can reduce the estimation time cost, it cannot address the problem of decreasing estimation efficiency in the case of high estimated channel dimensions. Moreover, the speed of tensor decomposition present in the algorithm decreases significantly when a large number of channels are estimated, which will greatly affect the estimation efficiency of the channel.

To address the problem of decreasing estimation efficiency due to excessive iteration steps in the PARAFAC-based algorithm, in this paper, we propose an accelerated bilinear least squares algorithm (ABALS) to solve the RIS-assisted MISO downlink communication channel estimation problem, and significantly improve the estimation efficiency. The main contributions of our work are as follows:

(1) We unfold the high-dimensional tensor involving unknown channels based on PARAFAC decomposition. Based on the tensor unfolded forms, we can transform the channel estimation problem into a cost function iterative optimization problem associated with the unknown channel.

(2) We propose an accelerated channel estimation algorithm. By introducing simpler iterative optimization constraints and linear interpolation, the channel can be estimated based on alternating optimization. The proposed algorithm can effectively accelerate the estimation procedure and accurately estimate the channel without degrading the estimation accuracy.

(3) In addition, we investigate the feasibility conditions and computational complexity of the ABALS algorithm and provide systematic recommendations for receivers to ensure the uniqueness of the channel estimation problem.

The rest of this paper is organized as follows: Section 2 derives the tensor model of the considered RIS-assisted MISO communication system and outlines the PARAFAC decomposition method for the channel. Section 3 proposes an accelerated bilinear least squares algorithm based on the ALS algorithm, discusses the feasibility conditions of the algorithm, and calculates the complexity of the algorithm. Section 4 provides and analyzes the simulation results. Conclusions are drawn in Section 5.

## 2. System and Signal Models

### 2.1. System Model

**Figure 1:**
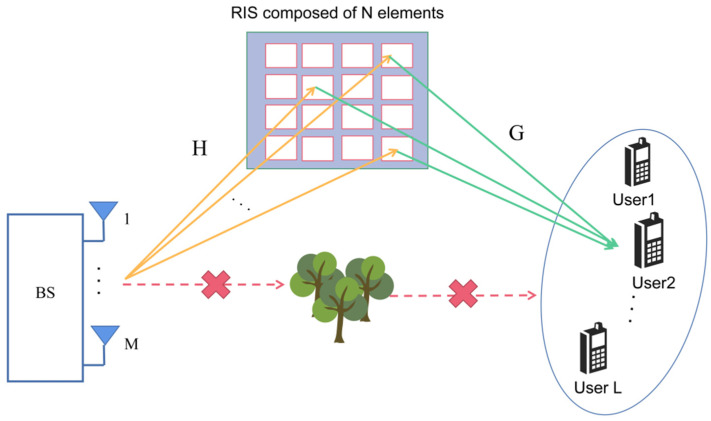
RIS-assisted multi-user MISO communication system.

As shown in Figure 1, we consider a RIS-assisted MISO system, where the BS is equipped with M antennas. There is a user community near the BS with L single-antenna mobile users. The direct channel paths between the BS and the mobile user community are blocked by obstacles. Since the propagation environment is not favorable, we assume that a passive RIS is deployed on the surface of a building close to the BS side. Signal transmission is carried out between the BS and the user community through RIS (In addition, our proposed method can also deal with the scenario where the direct link is not blocked. By turning off the RIS, we can easily estimate the direct channel through existing methods).

The RIS consists of N unit cells of equal small size, each capable of adjusting its reflection coefficient. We use the k-th feasible RIS phase configuration to transmit the signal, and during the T time slots, the received discrete-time signals for all mobile users can be represented by the matrix $Y_{k}\in {\mathbb{C}}^{L\times T}$ as follows:

(1) $$Y_{k}=GD_{k}\left(\Phi \right)HX+N_{k}$$

where $D_{k}\left(\Phi \right)≜\mathrm{diag}\left({\left[\Phi \right]}_{k,:}\right)$, and ${\left[\Phi \right]}_{k,:}$ denotes the k-th row of the K×N complex valued matrix Φ. Each row includes all feasible RIS phase configurations being usually selected from the low-resolution discrete set. Notations $G\in {\mathbb{C}}^{L\times N}$ and $H\in {\mathbb{C}}^{N\times M}$ denote the channels between the user community and the RIS and between the RIS and the BS, respectively. The signal $X\in {\mathbb{C}}^{M\times T}$ includes the BS transmission signal within T time slots. In addition, to avoid the problem of poor estimation accuracy during channel estimation, T>M must be maintained for effective estimation. Finally, the $N_{k}\in {\mathbb{C}}^{L\times T}$ denotes the additive Gaussian white noise matrix.

### 2.2. Preliminaries on the PARAFAC Decomposition

PARAFAC decomposition is an essential tool for data analysis in chemometrics, signal processing, and other applications [27]. We decompose the three-dimensional tensor $X\in {\mathbb{C}}^{I\times J\times K}$ into a sum of three rank-one tensors, and the decomposition result can be expressed as follows:

(2) $$X=\overset{R}{\underset{r=1}{\sum }}a_{r}\circ b_{r}\circ c_{r}$$

We define $A=\left[a_{1},...,a_{R}\right]$, $B=\left[b_{1},...,b_{R}\right]$, and $C=\left[c_{1},...,c_{R}\right]$, and then (2) can be written as:

(3) $$X=\overset{R}{\underset{r=1}{\sum }}{\left[A\right]}_{ir}{\left[B\right]}_{jr}{\left[C\right]}_{kr}$$

In addition, each dimension along the tensor can be partitioned into three different cuts, namely:

(4) $$X\left(i,:,:\right)=B\mathrm{diag}\left(A\right)C^{T}$$

(5) $$X\left(:,j,:\right)=A\mathrm{diag}\left(B\right)C^{T}$$

(6) $$X\left(:,:,k\right)=A\mathrm{diag}\left(C\right)B^{T}$$

We arrange the slices of the above to obtain three corresponding matrix expansion modes, as follows:

(7) $$X^{\left(1\right)}=\left(B\circ C\right)A^{T}$$

(8) $$X^{\left(2\right)}=\left(A\circ C\right)B^{T}$$

(9) $$X^{\left(3\right)}=\left(A\circ B\right)C^{T}$$

where ∘ represents the Khatri-Rao product, and $X^{\left(1\right)},\;X^{\left(2\right)},\;X^{\left(3\right)}$ can be viewed as horizontal, lateral, and frontal slices of the tensor X.

### 2.3. Signal Model

The acquisition of channel state information for channels G and H is a critical problem requiring resolution. We assume that these matrices are independent, and have identically distributed complex Gaussian terms, which are independent of each other. To simplify this estimation problem, the signal X in (1) is designed as an orthogonal pilot signal, i.e., $XX^{H}=I_{M}$. In the channel estimation period, we adopt the Φ with K different phase configurations, and the received training signal that removed the pilot signal can be defined as follows:

(10) $$Y_{k}X^{H}=GD_{k}\left(\Phi \right)H+N_{k}X^{H}$$

We define ${\tilde{Y}}_{k}=GD_{k}\left(\Phi \right)H$ as the noise-free wireless channel from the BS to the UE, ${\tilde{N}}_{k}=N_{k}X^{H}$ is the noise matrix after removing the pilot signal, and the matrix ${\tilde{Y}}_{k}$ can be viewed as the k-th frontal matrix slice of a three-dimension tensor according to the PARAFAC decomposition. We define $Z=H^{T}$, and the (l,m)-th entry of the noiseless received signal ${\tilde{Y}}_{k}$ can be obtained as:

(11) $${\left[{\tilde{Y}}_{k}\right]}_{l,m}=\overset{N}{\underset{n=1}{\sum }}{\left[G\right]}_{l,n}{\left[Z\right]}_{m,n}{\left[\Phi \right]}_{k,n}$$

Based on the tri-linearity of the PARAFAC decomposition, each ${\tilde{Y}}_{k}$ in (11), out of the K in total, can be represented by three different matrix forms. As shown in (12)–(14), we build the tensor $Y\in {\mathbb{C}}^{L\times M\times K}$ that contains all the matrices in the three dimensions, ${\tilde{Y}}_{k}$ matrices, so the tensor can be unfolded as mode 1, mode 2, and mode 3 [28], which can be expressed as:

(12) $$Y_{\alpha }≜\left(Z\circ \Phi \right)G^{T}\in {\mathbb{C}}^{\mathrm{KM}\times L}$$

(13) $$Y_{\beta }≜\left(\Phi \circ G\right)Z^{T}\in {\mathbb{C}}^{\mathrm{KL}\times M}$$

(14) $$Y_{\gamma }≜\left(G\circ Z\right)\Phi^{T}\in {\mathbb{C}}^{\mathrm{LM}\times K}$$

$Y_{\alpha }$, $Y_{\beta }$, and $Y_{\gamma }$ can be viewed as the products of a Khatri-Rao product and a single matrix. Based on this feature of the unfolding model, we can transform the estimation problem of the two unknown channels into two cost function optimization problems for decoupled estimation of the two channels.

## 3. Proposed PARAFAC-Based Channel Estimation

### 3.1. ABALS Channel Estimation

To efficiently estimate the channels G and H, inspired by the bilinear alternating least squares algorithm, which is envisioned as a key technique for high-dimensional low-rank decomposition, we propose the ABALS algorithm.

We suppose the three-dimensional matrix $\tilde{N}\in {\mathbb{C}}^{L\times M\times K}$ contains all additive Gaussian white noise matrices ${\tilde{N}}_{k}$, then the three-dimensional matrix $\tilde{Y}\in {\mathbb{C}}^{L\times M\times K}$ is written as:

(15) $$\tilde{Y}≜Y+\tilde{N}$$

Clearly, Y in (15) represents the noiseless version of $\tilde{Y}$, and by expanding $\tilde{Y}$ according to the form in (12)–(14), we obtain the expanded forms ${\tilde{Y}}_{\alpha }\in {\mathbb{C}}^{L\times \mathrm{MK}}$, ${\tilde{Y}}_{\beta }\in {\mathbb{C}}^{M\times \mathrm{LK}}$, and ${\tilde{Y}}_{\gamma }\in {\mathbb{C}}^{K\times \mathrm{ML}}$.

Similar to the ALS algorithm [26], we first generate an initialized channel matrix G and Z at random that satisfies an independent complex Gaussian distribution. When estimating each matrix, the other matrices should maintain their previous estimates until the cost function converges to the minimum value. To hasten the convergence of the algorithm, we compute a linear search process for linear interpolation of Z. The interpolation of the i-th iteration is designated as:

(16) $$G_{\mathrm{new}}^{\left(i\right)}={\hat{G}}^{\left(i-2\right)}+\rho ({\hat{G}}^{\left(i-1\right)}-\;{\hat{G}}^{\left(i-2\right)})$$

where ρ is the relaxation factor, denotingthe search direction at the i-th iteration, and ${\hat{G}}^{\left(i-1\right)}$ and ${\hat{G}}^{\left(i-2\right)}$ denote the (i−1)-th and (i−2)-th estimation values of the channel G. We choose a suitable step size, assuming ρ=i1α with α=3. Now, the residual error is calculated for the extrapolated matrices, as:

(17) $$\delta_{p}\left(i\right)={||{\tilde{Y}}_{\alpha }-\left(Z^{\left(i\right)}\circ \Phi \right){\left(G_{\mathrm{new}}^{\left(i\right)}\right)}^{T}||}_{F}^{2}$$

where ${||\cdot ||}_{F}$ denotes the matrix Frobenius norm. By computing the iteration function in (17) and comparing the cost function in two subsequent iterations, we may decide whether to apply linear interpolation in the iterative estimating process. When $\delta_{p}\left(i\right)\leq \delta_{p}\left(i-1\right)$, we let ${\hat{G}}^{\left(i\right)}={\hat{G}}_{\mathrm{new}}^{\left(i\right)}$, otherwise we let ${\hat{G}}^{\left(i\right)}={\hat{G}}^{\left(i-1\right)}$.

After determining the values of ${\hat{G}}^{\left(i\right)}$, by using the received signal provided in Equation (15) and its unfolded form, we estimate the channels G and H through optimizing the two cost functions iteratively. At the stage of optimizing the two functions, we start with the channel H. In the (i+1)-th iteration of the algorithm, the channel estimation result of H is obtained from the minimization of the following cost function:

(18) $${\hat{Z}}^{\left(i+1\right)}=\arg\underset{Z}{\min}{||{\tilde{Y}}_{\beta }-\left(\Phi \circ {\hat{G}}^{\left(i\right)}\right){\left({\hat{Z}}^{\left(i\right)}\right)}^{T}||}_{F}^{2}$$

The closed-loop solution of (18) is as follows:

(19) $${\hat{H}}^{\left(i+1\right)}={\left({\hat{Z}}^{\left(i+1\right)}\right)}^{T}={\left(\Phi \circ {\hat{G}}^{\left(i\right)}\right)}^{†}{\tilde{Y}}_{\beta }$$

where ${\left(\cdot \right)}^{†}$ stands for the matrix pseudo-inverse. Similarly, when estimating the channel G, we construct the estimated cost function based on mode 1 as follows:

(20) $${\hat{G}}^{\left(i+1\right)}=\arg\underset{G}{\min}{||{\tilde{Y}}_{\alpha }-\left({\hat{Z}}^{\left(i+1\right)}\circ \Phi \right){\left({\hat{G}}^{\left(i+1\right)}\right)}^{T}||}_{F}^{2}$$

The (i+1)-th estimate of the channel G can be obtained as follows:

(21) $${\left({\hat{G}}^{\left(i+1\right)}\right)}^{T}={\left({\hat{Z}}^{\left(i+1\right)}\circ \Phi \right)}^{†}{\tilde{Y}}_{\alpha }$$

When $\left|e_{\left(i+1\right)}-e_{\left(i\right)}\right|\leq \varepsilon$, the iteration terminates, where $e_{\left(i+1\right)}={||{\tilde{Y}}_{\alpha }-{\hat{Y}}_{\alpha }||}_{F}^{2}$ indicates the reconstruction error of the (i+1)-th iteration with ${\hat{Y}}_{\alpha }≜\left({\hat{Z}}^{\left(i+1\right)}\circ \Phi \right){\left({\hat{G}}^{\left(i+1\right)}\right)}^{T}$, and ε is a threshold parameter. When the iteration reaches convergence, the estimated channel matrices G and H are obtained. It should be noted that the scale ambiguity of the convergence point occurs when estimating the channels H and G. The accurate channel estimation results can be obtained by adequate normalization.

In comparison with ALS, the ABALS algorithm requires fewer iteration steps and maintains the same accuracy. It is worth noting that for each iteration step, due to the linear interpolation in ABALS and the extra calculation of the cost function, the calculation cost will increase, but fewer iteration steps will compensate for the increase of the calculation cost. In addition, the complexity of the constraint condition based on the cost function chosen in this paper is lower compared with the traditional constraint condition complexity, which will not cause a larger computational burden. Moreover, the ABALS algorithm can effectively solve the swamp problem that may occur in estimation in the non-degenerate case [29], and can significantly reduce the number of iterations in the algorithm. Therefore, the estimation speed of the ABALS algorithm is faster compared with that of the ALS algorithm. The ABALS algorithm can be summarized as follows (Algorithm 1).
**Algorithm 1.** Proposed Accelerated Bilinear Alternating Least Squares**Procedure** **Input**: Received tensor and the error threshold; set i=1; Randomly initialize ${\hat{G}}^{\left(i=0\right)}$, ${\hat{G}}^{\left(i=1\right)}$. **Output**: Estimates $\hat{H}$, $\hat{G}$. **begin**   **While** |e(i)−e(i−1)|>ε **do**    i=i+1;    1: Compute $\delta_{p}\left(i\right)$ from (17) with =i1α, and compare it with $\delta_{p}\left(i-1\right)$.    If${\;\delta }_{p}\left(i\right)\leq \delta_{p}\left(i-1\right)$,$\;{\hat{G}}^{\left(i-1\right)}=G_{\mathrm{new}}^{\left(i-1\right)}$;    else$\;{\hat{\;G}}^{\left(i-1\right)}={\hat{G}}^{\left(i-1\right)}$;    2: Update H using $\Phi ,G^{\left(i-1\right)},{\hat{Y}}_{\beta }$ by:$\;{\hat{H}}^{\left(i\right)}={\left({\hat{Z}}^{\left(i\right)}\right)}^{T}={\left(\Phi \circ {\hat{G}}^{\left(i-1\right)}\right)}^{†}{\tilde{Y}}_{\beta }$.    3: Update G using $\Phi ,Z^{\left(i\right)},{\hat{Y}}_{\alpha }$ by: ${\left({\hat{G}}^{\left(i\right)}\right)}^{T}={\left({\hat{Z}}^{\left(i\right)}\circ \Phi \right)}^{†}{\tilde{Y}}_{\alpha }$.    4: Repeat steps 1 to 3 until the loop termination condition is met.   **end** **end**

### 3.2. Feasibility Conditions

In order to guarantee that the algorithm can obtain accurate channel estimation results, it is essential to ensure that the two Khatri-Rao products $M_{1}=Z\circ \Phi \in {\mathbb{C}}^{\mathrm{KM}\times N}$ and $M_{2}=\Phi \circ G\in {\mathbb{C}}^{\mathrm{KL}\times N}$ columns appearing in the algorithm are full rank, which means that KM≥N and KL≥N must be satisfied. Combined with the two inequalities, this implies that min(KM,KL)≥N can be equivalently expressed as:

(22) Kmin(M,L)≥N

Note that satisfying Condition (22) does not guarantee the uniqueness of the ALS algorithm and the ABALS algorithm estimates [30]. We denote the Kruskal rank of the matrix A as $k_{A}$, and any k columns in A are linearly independent and the Kruskal rank is always less than or equal to the regular matrix rank. If A is of full column rank, then it is also of full Kruskal rank. The identifiability theorem based on the PARAFAC model in [30] can be proven if:

(23) $$k_{G}+k_{Z}+k_{\Phi }\geq 2N+2$$

Then, (G,Z,Φ) is unique in terms of alignment and scaling, and the relationship is shown as follows:

(24) $$\hat{G}=G\Pi \Delta_{1},\hat{\Phi }=\Phi \Pi \Delta_{2},\hat{Z}=Z\Pi \Delta_{3}$$

where Π is an N×N permutation matrix, and $\Delta_{1,2,3}$ are the diagonal scaling matrix of N×N, satisfying:

(25) $$\Delta_{1}\Delta_{2}\Delta_{3}=I_{N}$$

According to the considered channel model, channels G and H are both full-rank matrices, and Condition (23) is transformed into:

(26) min(L,N)+min(M,N)+min(K,N)≥2N+2

To obtain accurate CSI, it is necessary to ensure that the number of antennas is more than or equal to the number of RIS elements, and the number of users should be less than the number of antennas. Additionally, to ensure the acceleration of the proposed algorithm, the number of pilots should be less than half of the number of RIS elements, i.e., $P\leq \frac{N}{2}$.

From the above-mentioned feasibility conditions, it is clear that the feasibility conditions are not satisfied when the number of RIS unit elements is greater than the number of antennas or mobile users of BS. To resolve this conflict, the elements of RIS are divided into non-overlapping sub-cell groups. Each element exists in only one cell group, and the number of RIS cell elements in the divided cell group does not exceed the number of mobile users and the number of antennas. At this time, we can apply the two algorithms to estimate the channel of each sub-cell group and reconstruct the combination of the estimated results back to the required channel estimation results.

### 3.3. Computational Complexity

Here, we analyze the computational complexity of our proposed PARAFAC-based accelerated bilinear least squares algorithm, where the main computational task is the iterative algorithm, and the computational complexities associated with steps 2 and 3, which are the two left pseudo-inverses computed for each iteration, where the computational complexity of step 2 is $O\left(N^{2}\left(\mathrm{KM}+L\right)+\mathrm{NMLK}\right)$, and the computational complexity of step 3 is $O\left(N^{2}\left(\mathrm{KL}+M\right)+\mathrm{NMLK}\right)$. The main complexity of the algorithm is $\left(N^{2}\left(\mathrm{KM}+\mathrm{KL}+M+L\right)+2\mathrm{NMLK}\right)$. The complexity of the two-timescale channel estimation proposed by [13] mainly lies in the vector-matrix multiplication and the iterative solution of the channel, and its complexity is $O\left(N^{2}+\mathrm{MT}\right)$. The complexity of the LS algorithm mainly lies in the solution of the matrix pseudo-inverse and the multiplication, and its computational complexity is $O\left(\mathrm{MT}\left(T^{2}+T+L\right)\right)$. Furthermore, the complexity of the ALS algorithm is similar to the ABALS algorithm. It is clear that the complexity of the two-timescale channel estimation and the LS algorithm is very much affected by T. The ABALS algorithm removes the transmission signal X before the estimation, which means the complexity of our algorithm is not affected by the transmission signal.

## 4. Numerical Results

In this section, we provide several sets of simulation results to evaluate the performance of the proposed ABALS channel estimation method, while comparing the ALS algorithm proposed in [26]. The attribution of channel estimation accuracy normalized mean square error (NMSE) is obtained as follows:

(27) $$\mathrm{NMSE}\left(\hat{H}\right)=\frac{1}{P}\overset{P}{\underset{p=1}{\sum }}\frac{{||H^{\left(p\right)}-{\hat{H}}^{\left(p\right)}||}_{F}^{2}}{{||H^{\left(p\right)}||}_{F}^{2}}$$

where $H^{\left(p\right)}$ is the channel between BS-RIS estimated at the p-th run, and p represents the number of Monte Carlo simulations. The same definition is applicable to the estimated RIS-UE channel. For the scale ambiguity in the algorithm, we can remove it by normalizing the first column of the channel matrix. Φ is set to be the discrete Fourier transform matrix, satisfying $\Phi^{H}\Phi =I_{N}$ at the period of estimating channels H and G. All NMSE curves are obtained after averaging over 2000 independent Monte Carlo channels, with a threshold of $\varepsilon =10^{-6}$ for each independent simulation.

**Figure 2:**
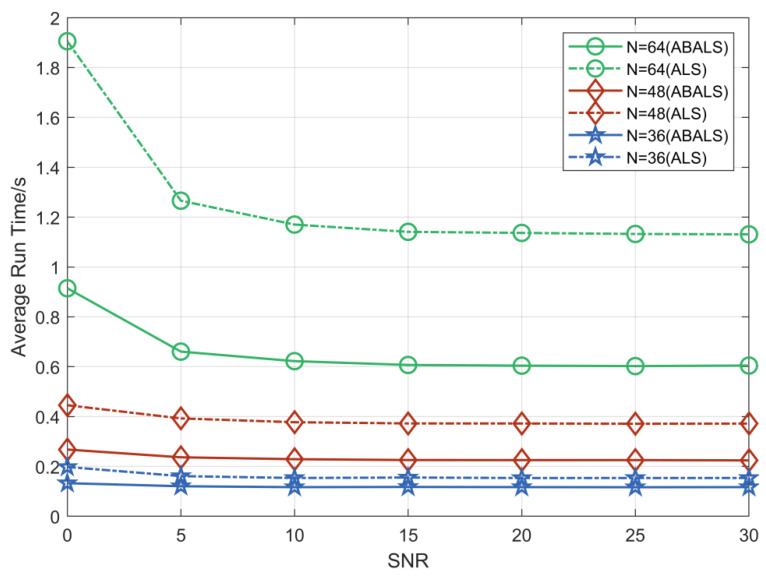
Average runtime of ABALS and ALS algorithms.

**Figure 3:**
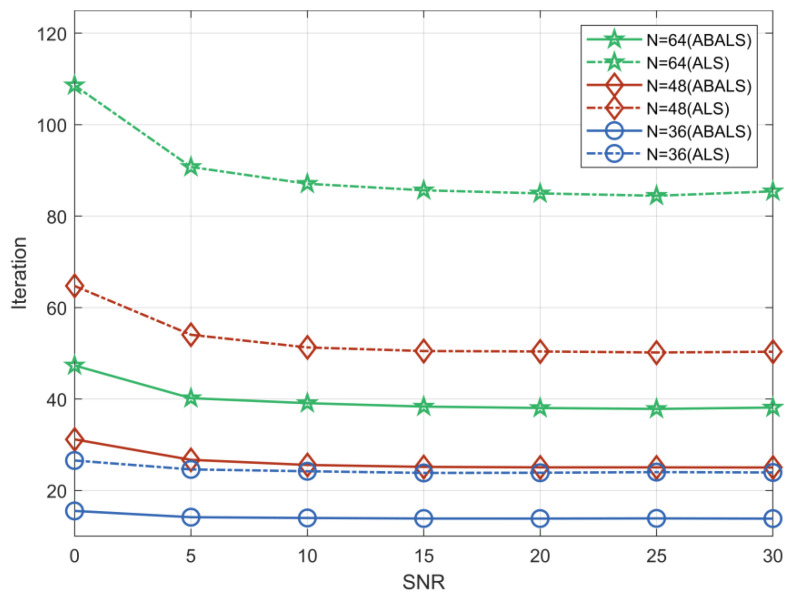
Number of iterations to convergence of the ABALS and ALS algorithms.

We evaluated the average running time and the number of iterations required for the ABALS algorithm and the ALS algorithm for different numbers of RIS elements. As shown in Figure 2 and Figure 3, we set the system parameters M=64, L=32, K=16, and N=32, 48, 64. Compared with the ALS algorithm, the iteration time of the ABALS algorithm was reduced by about 50%. In particular, the average running time as well as the number of iterations of the ABALS algorithm were significantly reduced when N=64, since the linear search used in the ABALS algorithm effectively mitigates the swamp phenomenon present in the ALS algorithm and improves the channel estimation speed. In addition, the running time of the ABALS algorithm gradually leveled off when the signal-to-noise ratio (SNR) was over 10 dB because the PARAFAC decomposition process can effectively reduce the impact of noise on the estimation, which indicates that the ABALS algorithm has better noise immunity. In terms of the number of iterations, the number of iterations required by the ABALS and the ALS algorithms grew with the increase of the number of RIS elements, and there was a more obvious change in the convergence speed for different numbers of RIS elements in the low SNR range. In summary, the ABALS algorithm has a higher estimation speed with fewer iteration steps, and the algorithm can effectively combat noise.

**Figure 4:**
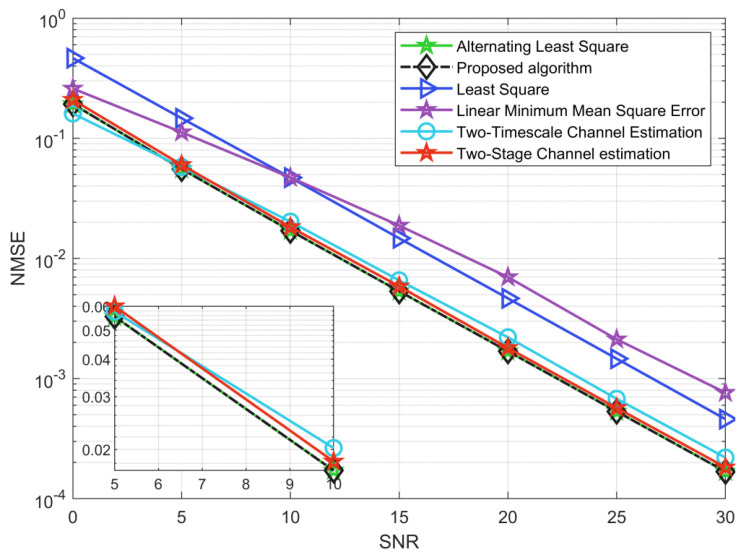
NMSE for the cascaded channel between the different algorithms.

By setting M = 64, L = 32, N = 48, and K = 16, Figure 4 depicts the NMSE performance comparison of the ALS algorithm [26], LS algorithm, LMMSE algorithm [31], two-timescale channel estimation [13], and two-stage channel estimation [18]. The results show that the ABALS algorithm is very similar to the ALS algorithm in terms of accuracy and outperformed the other four algorithms. In particular, along with the increase in SNR, the performance gap between the ABALS algorithm and the LMMSE algorithm is increasing. This is because our proposed algorithm is less restrictive. Additionally, the ABALS algorithm can obtain a stable gain of 5 dB compared with the LS algorithm, since the proposed ABALS algorithm achieves channel estimation by decoupling the channels H and G instead of estimating $\theta =\mathrm{vec}\left(H^{T}\circ G\right)$. In addition, our algorithm has similar accuracy to the two-timescale channel estimation method, and when SNR∈[5,30], the ABALS algorithm outperformed the two-timescale estimation method. The proposed ABALS algorithm has better performance compared with two-stage channel estimation because the ABALS algorithm is not affected by the pilot signal and can obtain better estimation results.

**Figure 5:**
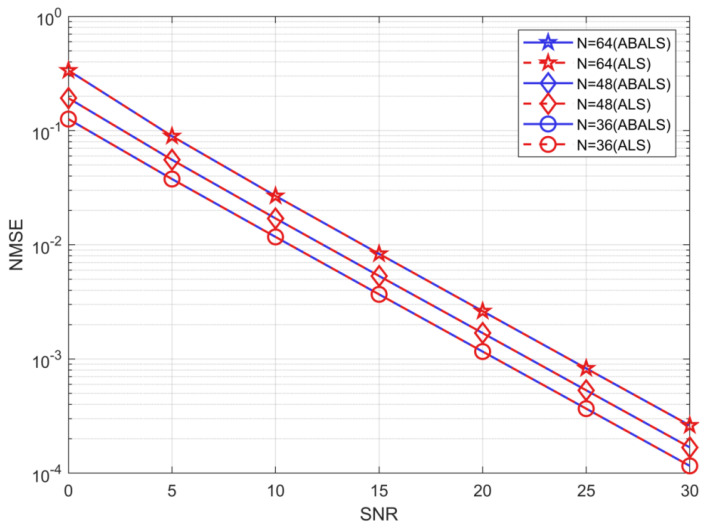
NMSE for the cascaded channel.

As shown in Figure 5, the system parameters are M=64, L=32, K=16, and N ={32, 48, 64}. The results depict the algorithmic performance of the cascaded channel estimation ABALS algorithm and ALS for NMSE performance for different numbers of RIS elements. The proposed ABALS and ALS algorithms have similar performance with different N, because the two algorithms set the same iteration termination threshold. The proposed ABALS algorithm speeds up the iteration process, which makes the algorithm reach the convergence threshold earlier. The NMSE of the ABALS algorithm is close to $10^{-1}$ when the SNR =0 dB. The estimation error decreases sharply as the SNR increases, and the estimated NMSE decreases by three orders of magnitude when the SNR =30 dB compared with SNR =0 dB. From the above results, it is clear that the proposed ABALS algorithm can achieve similar performance to the ALS algorithm and have high estimation accuracy at a low SNR. In addition, since the accuracy of the two algorithms is very close, in the subsequent analysis, we only analyze the performance of the ABALS algorithm.

**Figure 6:**
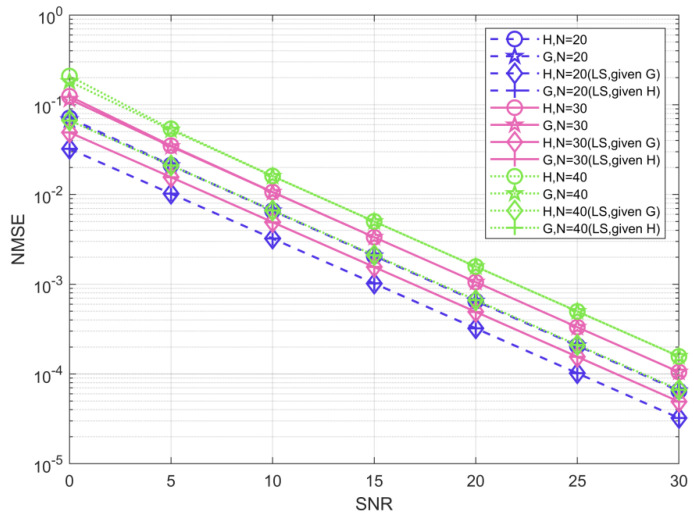
NMSE of the estimated channels H and G.

Figure 6 shows the NMSE performance of the ABALS algorithm for channel H and G estimation with different numbers of RIS elements. The parameters used to obtain this figure were M=48, L=48, K=10, and N={20, 30, 40}. We assume that one channel is known and estimate the other channel based on LS, and the obtained estimation results can be considered as the best accuracy of the alternating least squares algorithm. As shown in the figure, the performance gap between the ABALS algorithm and the best accuracy of the ALS algorithm is about 6 dB. Since the algorithm uses alternate optimization to obtain the CSI, there is a certain error during two-channel estimation, which is unavoidable. The ABALS algorithm has a high and similar accuracy for both channels, indicating that the proposed algorithm can perform efficient estimation for both channels. It is notable that when M is equal to L, the channels H and G have the same dimensions, which makes the estimation accuracy of the two channels very similar. However, when the channels H and G have different dimensions, there is a difference in the estimation accuracy of the two channels, which is caused by the fact that the channel with a larger dimension requires more parameters to be estimated. In addition, the performance loss keeps increasing as the number of RIS elements increases. Larger N leads to a larger number of rows and columns for H and G, respectively, and an increase in the channel parameters to be estimated, which leads to a decrease in the estimation accuracy. To reduce the NMSE of the algorithm, it requires higher training pilots.

**Figure 7:**
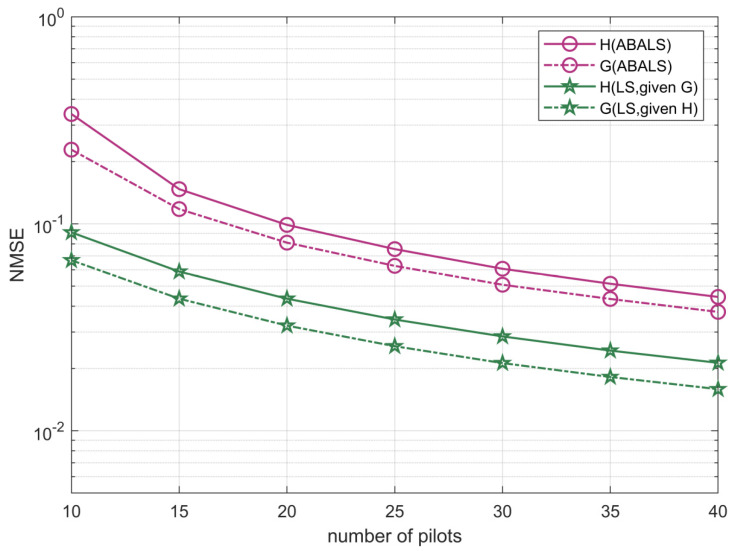
NMSE performance of the ABALS channel estimation versus the number of pilots.

The effect of the number of pilots K on the NMSE performance is depicted in Figure 7 by setting M=64, L=48, N=48, and SNR =0 dB. The results show that the number of training pilots had a significant effect on the performance of the proposed algorithm. The increase in the number of pilots overhead reduced the estimation error. This happens because as the number of pilot increases, the training set increases, and this can make the results more accurate. When the number of transmission pilots is less than 20, the algorithm performance quickly enhances. Moreover, when the number of transmission pilots is greater than 20, the algorithm performance improvement becomes slower, since more transmission pilots will cause a higher computational complexity. According to the feasibility condition, the number of pilots should be smaller than the number of elements of RIS to ensure higher estimation accuracy and lower complexity.

## 5. Conclusions

For RIS-assisted MISO communication systems, we have proposed an accelerated bilinear least squares algorithm. This method enables separate estimation of the BS-RIS and RIS-UE channels at the receiver side. We computed the linear interpolation of the BS-RIS channel, introduced optimization constraints based on the cost function, selected the appropriate step size for cases that satisfy the constraints, and effectively extrapolated the iterations. As a result, the algorithm can efficiently accelerate the estimation procedure, without losing accuracy. Furthermore, we conducted a feasibility analysis and calculated the complexity of the algorithm. The simulation results show that, compared to the ALS method, the ABALS algorithm estimated the BS-RIS channel and the RIS-UE channel more quickly and with fewer iteration steps, while maintaining a relatively similar accuracy.

We can see that the algorithm proposed in this paper can accelerate the channel estimation procedure. However, this paper only studied the system of single-antenna users and did not discuss the situation of multi-antenna users. Therefore, in future work, research can be conducted to propose a parallel decomposition channel estimation scheme adapted to multi-antenna users. In addition, if we want to achieve fast channel estimation in a MIMO system, simulation results have shown that it is optional to use a parallel decomposition scheme.

## Figures and Tables

**Table 1:** Summary of previous works.

Antenna Setup	Representative Work
RIS-assisted MIMO	Cascade channel estimation based on sparse matrix factorization and complementation [14].
	Cascade channel estimation based on atomic parametric minimization [15].
	Cascade channel estimation based on deep learning [20].
	Separate channel estimation using the on/off reflection model at RIS [11].
	Separate channel estimation based on an iterative algorithm [12].
	Separate channel estimation based on the tensor model and its algebraic structure [24].
RIS-assisted MISO	Cascade channel estimation based on a two-timescale channel estimation framework and a coordinate decent-based algorithm [13].
	Cascade channel estimation based on an LMMSE estimator [17].
	Separate channel estimation based on an active sensor-aided algorithm [16].
	Separate channel estimation based on a vector-based approximate message-passing algorithm [25].
RIS-assisted SISO	Cascade channel estimation based on the channel correlation [9,10].

## Data Availability

Not applicable.
